# Glucagon-like peptide-1 (GLP-1) raises blood-brain glucose transfer capacity and hexokinase activity in human brain

**DOI:** 10.3389/fnene.2013.00002

**Published:** 2013-03-27

**Authors:** Michael Gejl, Susanne Lerche, Lærke Egefjord, Birgitte Brock, Niels Møller, Kim Vang, Anders B. Rodell, Bo M. Bibby, Jens J. Holst, Jørgen Rungby, Albert Gjedde

**Affiliations:** ^1^Department of Biomedicine - Pharmacology, Aarhus UniversityAarhus, Denmark; ^2^Department of Clinical Biochemistry, Aarhus University HospitalAarhus, Denmark; ^3^Medical Research Laboratory M, Aarhus University HospitalAarhus, Denmark; ^4^Department of Nuclear Medicine and PET-Center, Aarhus University HospitalAarhus, Denmark; ^5^Department of Biostatistics, Aarhus UniversityAarhus, Denmark; ^6^Department of Biomedical Sciences, Panum Institute, University of CopenhagenCopenhagen, Denmark; ^7^Department of Neuroscience and Pharmacology, Panum Institute, University of CopenhagenCopenhagen, Denmark; ^8^Department of Radiology and Radiological Science, Johns Hopkins UniversityBaltimore, MD, USA; ^9^Department of Neurology and Neurosurgery, McGill UniversityMontréal, QC, Canada

**Keywords:** glucagon-like peptide -1, hypoglycemia, hyperglycemia, blood-brain barrier, cerebral metabolic rate for glucose, Michaelis-Menten, cerebral glucose transport

## Abstract

In hyperglycemia, glucagon-like peptide-1 (GLP-1) lowers brain glucose concentration together with increased net blood-brain clearance and brain metabolism, but it is not known whether this effect depends on the prevailing plasma glucose (PG) concentration. In hypoglycemia, glucose depletion potentially impairs brain function. Here, we test the hypothesis that GLP-1 exacerbates the effect of hypoglycemia. To test the hypothesis, we determined glucose transport and consumption rates in seven healthy men in a randomized, double-blinded placebo-controlled cross-over experimental design. The acute effect of GLP-1 on glucose transfer in the brain was measured by positron emission tomography (PET) during a hypoglycemic clamp (3 mM plasma glucose) with ^18^F-fluoro-2-deoxy-glucose (FDG) as tracer of glucose. In addition, we jointly analyzed cerebrometabolic effects of GLP-1 from the present hypoglycemia study and our previous hyperglycemia study to estimate the Michaelis-Menten constants of glucose transport and metabolism. The GLP-1 treatment lowered the vascular volume of brain tissue. Loading data from hypo- to hyperglycemia into the Michaelis-Menten equation, we found increased maximum phosphorylation velocity (*V*_max_) in the gray matter regions of cerebral cortex, thalamus, and cerebellum, as well as increased blood-brain glucose transport capacity (*T*_max_) in gray matter, white matter, cortex, thalamus, and cerebellum. In hypoglycemia, GLP-1 had no effects on net glucose metabolism, brain glucose concentration, or blood-brain glucose transport. Neither hexokinase nor transporter affinities varied significantly with treatment in any region. We conclude that GLP-1 changes blood-brain glucose transfer and brain glucose metabolic rates in a PG concentration-dependent manner. One consequence is that hypoglycemia eliminates these effects of GLP-1 on brain glucose homeostasis.

## Introduction

Brain energy metabolism almost exclusively depends on a steady supply of circulating glucose. The adequacy of the glucose delivery in turn depends on the glucose concentration in plasma. Therefore, hypoglycemia can induce brain dysfunction and hypoglycemic symptoms (Maran et al., 1995). Glucagon-like peptide-1 (GLP-1) is produced in the brain by neurons in the nucleus of the solitary tract where it acts as a neuropeptide, as well as in the gut. GLP-1 has beneficial effects on both peripheral (Holst, 2007) and cerebral glucose homeostasis (Lerche et al., 2008; Gejl et al., 2012a). Central GLP-1 signaling is linked to the control of blood glucose concentrations (D'Alessio et al., 2005), and studies reveal extrapancreatic effects of GLP-1 (Vella and Rizza, 2004; Bak et al., 2011; Gejl et al., 2012b).

Glucose is supplied to the brain by the stereospecific and non-energy-demanding mechanism of facilitated diffusion, mediated by the GLUT1 transporter at the blood-brain barrier (BBB) (Gjedde, 1983). The equation of Michaelis and Menten (1913) describes the relationship between glucose transport across the BBB and the glucose concentration in plasma (Crone, 1965) and it is in turn consistent with an inverse relation between the glucose extraction and the glucose concentration, such that the glucose extraction fraction falls in hyperglycemia (Gjedde, 1981; Hasselbalch et al., 2001) and rises in hypoglycemia (Blomqvist et al., 1991). As insulin has no direct effect on blood-brain glucose transfer, it remains uncertain how glucose transport across the BBB is regulated by factors other than the concentration of glucose (Hasselbalch et al., 1999) and the transport capacity of the GLUT1 transporter.

Previously, in hyperglycemia, we showed that GLP-1 lowers the glucose concentration in brain tissue (*C*_tissue_) in association with increased net clearance of glucose across the BBB (Gejl et al., 2012a). With an unaltered transporter transport capacity (*T*_max_) of the GLUT1 transporter in the BBB, we reasoned that the most likely explanation is an increase of the maximum velocity of phosphorylation (*V*_max_), but the findings were not conclusive, as we observed the cerebral metabolic rate for glucose (*CMR*_glc_) to be increased in several regions but not in the brain as a whole. This hypothetical increase of *V*_max_, leading to less increase of *C*_tissue_ in response to the elevated plasma glucose, could contribute to GLP-1's neuroprotective effect at normal and higher glucose levels. Net glucose transfer is mediated by GLUT1 transporters in glial membranes as well as GLUT3 transporters in neuronal membranes, in addition to the GLUT1 transporters in the two membranes of the microvascular endothelium (Gjedde and Christensen, 1984; Simpson et al., 2007).

Glucose delivery falls when plasma glucose (PG) concentrations decline, but it is uncertain how GLP-1 affects the transport through the multiple membranes of the BBB and the brain cells in hypoglycemia, which limits the blood-brain glucose exchange. The main effect of hypoglycemia on blood-brain glucose delivery is a reduction of the unidirectional and net glucose fluxes across the BBB, as described by the Michaelis-Menten formalism. A hypothetical effect of GLP-1 directly on the magnitude of *V*_max_ would be expected to increase the phosphorylation rate even at hypoglycemia and the accompanying low tissue glucose level, because of the very high affinity (i.e., low *K*_*M*_) of hexokinase. A further effect of GLP-1 on blood-brain glucose transfer and glucose metabolism in the brain would accentuate the effects of hypoglycemia, with increasingly adverse results due to the low glucose.

Hypoglycemia is associated with reduced brain glucose content whether subjects remain aware or unaware of the change. To test the prediction that GLP-1 exacerbates the effect of hypoglycemia, we measured the transport and metabolism of the glucose tracer ^18^F-fluoro-2-deoxy-glucose (FDG) by positron emission tomography (PET) in the hypoglycemic state with and without the presence of GLP-1. We then pooled the findings with data obtained in hyperglycemia with and without GLP-1 (Gejl et al., 2012a), to reveal changes of the maximum phosphorylation and transport capacities (*V*_max_, *T*_max_) and the transport affinity of hexokinase (*K*_*M*_) and GLUT1 (*K*_*t*_).

## Materials and methods

Seven non-smoking, healthy, Caucasian males with a mean age of 22.9 ± (SD) 1.9 years and a mean body mass index of 22.7 ± 2.7 kg m^−2^ participated in the study. They had a normal physical examination, no history of diabetes or cardiovascular disease, and received no medication.

The study was conducted in accordance with the Declaration of Helsinki and the protocol was approved by the official Regional Science Ethics Committee of County Aarhus. All participants received both oral and written information and signed an approved informed consent form before entering the study.

The study proceeded as a randomized, double-blinded, placebo-controlled, crossover study. Each subject was studied twice in random order with GLP-1 and placebo infusion. PET sessions were separated by an interval of 2–6 weeks. Both sessions commenced at 9.00 h after an overnight fast. The subjects were instructed not to exercise 24 h prior to the sessions. Subjects were placed in bed and two catheters were inserted for infusion of clamp hormones and for the infusion of GLP-1 or placebo. A third catheter was placed in an arterialized (heated) dorsal hand vein for blood sampling. An arterial catheter was placed in the radial artery of the right arm in order to draw blood samples for measuring input radioactivity during PET.

A stepwise hypoglycemic pancreatic-pituitary clamp was performed according to principles previously described (Degn et al., 2004; Lerche et al., 2008; Gejl et al., 2012a). In brief somatostatin (Ferring GmbH, D-24109 Kiel) was infused at a rate of 50 μ g/min to suppress the endogenous insulin, glucagon, growth hormone (GH) and GLP-1 production. Human glucagon (Glucagen, Novo Nordisk A/S, Copenhagen, Denmark) 0.6 ng/kg/min and GH (Genotropin Miniquik 0.2 mg, Pfizer ApS, Ballerup, Denmark) 2 ng/kg/min were infused with the aim of maintaining near basal levels (0–360 min). Insulin (Actrapid, Novo Nordisk A/S, Copenhagen, Denmark) was infused at a rate of 0.8 mU/kg/min. Glucose (200 g/l) was infused at a variable rate to initially clamp PG at 4.5 mM (0–150 min). From 150 to 180 min, PG was lowered to 4.0 mM and maintained at this level until 210 min. From 210 min to 240 min PG was lowered to 3.5 mM and maintained at this level until 270 min. From 270 min to 300 min PG was lowered to a nadir of 3.0 mM and maintained at this level during PET (300–360 min). GLP-1 or placebo infusion was initiated at time 60 min and maintained during the entire session.

The subjects received either intravenous synthetic GLP-1 (7–36 amide) or placebo at a rate of 1.2 pmol/kg/min (60–360 min) (Toft-Nielsen et al., 1999). Recombinant human GLP-1 (7–36 amide) from BioNebraska Inc., Lincoln, NE (USA) was tested and found positive for sterility and negative for bacterial endotoxins before use. It was dissolved in a sterile buffer containing 600 mg of acetic acid, 50.7 g of mannitol and sterile water added up to 1000 g and had a pH of 4.5. The concentration of GLP-1 (7–36 amide) was 1 mg/ml and it was stored frozen in vials of 0.25 ml. The test solution consisted of 0.25 ml GLP-1, 20 ml of human albumin “ZLB” 5% and sodium chloride (9 g/l) to yield 100 ml of solution. The placebo solution consisted of the above mentioned buffer solution containing human albumin and saline. One subject experienced transient nausea and vomiting during GLP-1 infusion with onset at 20 min of infusion, lasting approximately 1½ h. Another subject experienced headache during placebo infusion after 2 h of infusion, lasting 1 h.

PG was measured in duplicate every 10 min until it reached 3.0 mM and then every fifth minute. Blood for measuring insulin, c-peptide, glucagon, GLP-1 (total and intact), GH, free fatty acids (FFA), cortisol and ghrelin was drawn every 30 min. Blood for measuring epinephrine and norepinephrine was drawn at 0, 150, and 240 min and every 30 min during the PET scan. Arterial blood for measuring input radioactivity was drawn at predetermined intervals during PET (12 × 5 s, 8 × 30 s, and 8 × 300 s).

### Hyperglycemia study

We incorporated the observations of cerebrometabolic effects of GLP-1 in hyperglycemia, presented by Gejl et al. (2012a).

### Assays

Assays were described previously (Lerche et al., 2008). Arterial input samples were analyzed on a *Packard radioactivity analyzer* immediately after each PET session.

### MRI

A high resolution T1-weighted MR was acquired for each subject with a 3.0 T Signa Excite GE Magnet using a 3DIR-fSPGR sequence.

### PET

FDG was used as a tracer for brain glucose uptake and was produced in-house according to the Drug Master File, Danish national marketing authorization number 2165. We used the whole-body PET EXACT HR47 (Siemens Medical, Knoxville, USA) with a 15 cm field-of-view and an acquisition capacity of 47 transaxial planes with a spatial resolution of 4–5 mm at the center of the field of view. All PET data were acquired in 3D mode. A 15-min transmission scan was performed to correct photon attenuation. Five hours after clamp start and 4 h after the GLP-1 or placebo infusion was started, a bolus of 200 MBq of FDG in 10 ml saline was injected intravenously over 10 s. Dynamic acquisition commenced at the beginning of tracer injection and continued for 45 min to acquire 23 frames (6 × 30 s, 7 × 1 min, 5 × 2 min, and 5 × 5 min).

### Image analysis

PET and MR-images were co-registered and entered in Talairach space.

Regional tissue time-activity-curves for FDG uptake were extracted for total cerebral gray matter, cerebral cortex, thalamus, striatum, cerebellar cortex, brainstem, and white matter.

### Kinetic analysis

Using a 3-compartment model, we obtained values of K^*^_1_ (unidirectional clearance where symbols marked with asterisk indicate FDG), k^*^_2_ (efflux rate constant), k^*^_3_ (phosphorylation rate constant) and *V*_*p*_ (volume of tissue occupied by intravascular blood) for FDG in each region-of-interest. The rate of dephosphorylation and the absolute quantity of FDG were both considered negligible during the scanning period. In the use of FDG to trace glucose metabolism, the “lumped constant” is a necessary isotope correction factor. In the model, the lumped constant is the ratio between the net uptakes of FDG and glucose which depends on the transport across the BBB and the phosphorylation of both. This relationship causes the lumped constant to increase in hypoglycemia compared to normo- or hyperglycemia. In the competition between native glucose and FDG in the brain, both transport across the BBB (K^*^_1_, k^*^_2_) and phosphorylation by hexokinase (k^*^_3_) obey the Michaelis-Menten equation, which yields fixed ratios of transport clearances (τ = K^*^_1_/*K*_1_) and phosphorylation rates (ϕ = k^*^_3_/*k*_3_) for the tracer (marked by asterisk) and native glucose (no asterisk) (Kuwabara et al., 1990). By substituting transfer constants of FDG for those of glucose, using the constants τ and ϕ, we determined the lumped constant directly for each subject in each region-of-interest as described previously (Kuwabara et al., 1990), using the values of τ = 1.48 and ϕ = 0.39 (Hasselbalch et al., 1998).

The net clearance of FDG is then (Gjedde, 1982)

$$K^{*}=K_{1}^{*}k_{3}^{*}/\left(k_{2}^{*}+k_{3}^{*}\right)$$

and the unidirectional glucose flux from blood into brain is calculated from the unidirectional FDG clearance as,

$$J_{\text{glc}}=K_{1}^{*}\;C_{a}/\tau ,$$

where *J*_glc_ is the unidirectional flux of glucose from blood to brain, *C*_a_ is the arterial steady state PG concentration (300–360 min), and symbols marked with asterisk indicate FDG.

The unidirectional clearance of glucose is by definition

$$K_{\text{1glc}}=K_{1}/\tau$$

With these constants the lumped constant has been shown to equal,

$$LC=\varphi +\left(\tau -\varphi \right)\left(k_{3}^{*}/\left(k_{2}^{*}+k_{3}^{*}\right)\right)$$

and the net clearance of glucose by definition is (Gjedde, 1982)

$$K=K^{*}/LC$$

such that the net cerebral metabolic rate for glucose is calculated from the net FDG clearance as,

$$CMR_{\text{glc}}=K^{*}\;C_{a}/LC,$$

and the cerebral tissue glucose concentration *C*_tissue_ is

$$C_{\text{tissue}}=\tau \left(J_{\text{glc}}-CMR_{\text{glc}}\right)/k_{2}^{*}.$$

From the equation of Michaelis and Menten (1913), the relationship between cerebral metabolic rate and *C*_tissue_ is defined as,

$$CMR_{\text{glc}}=V_{\text{max}}\times C_{\text{tissue}}/\left(K_{M}+C_{\text{tissue}}\right)$$

where *V*_max_ is the maximal phosphorylation velocity and *K*_*M*_ is the half-saturation-concentration of glucose.

The relationship between glucose transport across the BBB and glucose concentration in plasma likewise is defined as:

$$\text{Unidirectional flux}\left(J_{\text{glc}}\right)=T_{\text{max}}\times C_{a}/(K_{t}+C_{a})$$

where *T*_max_ is the maximal transport capacity and *K*_*t*_ is the half-saturation-concentration of plasma glucose.

### Calculations and statistics

The PET-data were analysed using a linear mixed effects model with subject and all interactions involving subject, including the interaction between subject and area and the interaction between subject and treatment, as random effects. Treatment (GLP-1 vs. placebo), region, and the interaction between the two were included in the analysis as fixed effects. To compare the two treatment groups regarding circulating hormones and metabolites a similar statistical model was used with time instead of region. In the analysis of the hypoglycemia study vs. the hyperglycemia study, we modified the linear mixed effects model to include an extra systematic factor (PG: hypoglycemia/hyperglycemia) along with all interactions between PG, treatment, and region as fixed effects. Significant differences were demonstrated with a significance level of *P* < 0.05. The statistical software used was GraphPad Prism (GraphPad Software, San Diego, CA, USA) and Stata (StataCorp LP, College Station, Texas, USA) Data are presented as mean ± SD. The study was planned as preliminary exploration and thus the number of subjects that entered the study did not derive from a power calculation.

All measurements corresponding to a single patient were excluded from the analysis due to values of *C*_tissue_ that were two orders of magnitude larger than for the other patients. Michaelis-Menten curves were fitted to the pooled data from the hypo- and hyperglycemia studies in each region using non-linear regression and taking into account that all patients were given both GLP-1 and placebo by including a random subject effect for each coefficient in the Michaelis-Menten model. The rate constants (*K*_*M*_ and *K*_*t*_) did not vary significantly with treatment in any region (all *p*-values above 0.17) and therefore a model with only one rate constant for each region was used to increase numerical stability. The data were analysed using nlme in R 2.15.0 [R Development Core Team (2012). R: A language and environment for statistical computing. R Foundation for Statistical Computing, Vienna, Austria. ISBN 3-900051-07-0, URL http://www.R-project.org/]. We used the in-house program iFit (www.liver.dk/ifit.html) for kinetic modeling.

## Results

**Figure 1:**
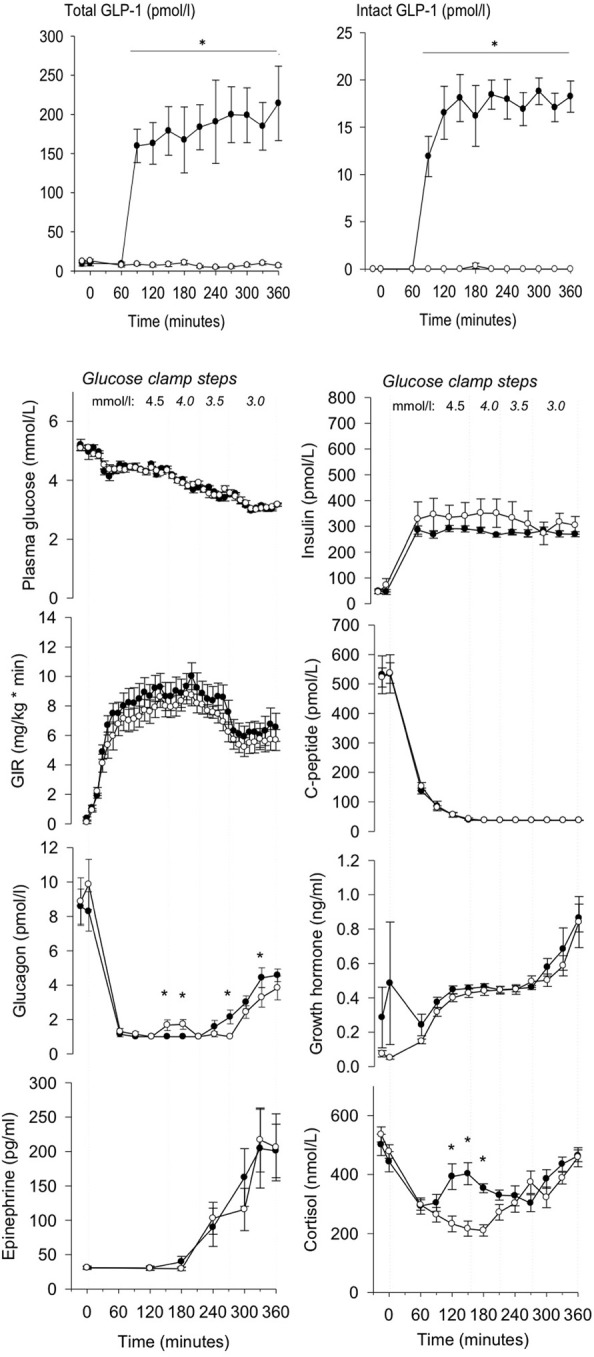
**Total, intact GLP-1 concentrations, plasma glucose, insulin, glucose infusion rate (GIR), C-peptide, glucagon, growth hormone, epinephrine and cortisol during GLP-1 (black dot) and placebo (white dot) infusion in different glucose clamp steps.** GLP-1 infusion was initiated at *t* = 60 min. PET was performed from 300 to 360 min. Data are mean ± SEM. ^*^P < 0.05.

The infusion of GLP-1 at a constant rate of 1.2 pmol/kg/ min resulted in pharmacologically relevant plasma concentrations of the intact hormone (Toft-Nielsen et al., 1999) (all plasma hormones and metabolites are depicted in Figure 1). During PET, the PG concentrations remained at 3.06 ± 0.08 (GLP-1) and 3.09 ± 0.07 (placebo) mM, *P* = 0.55 for the treatment effect with similar glucose infusion rates (6.27 ± 2.7 vs. 5.58 ± 2.15 mg/kg/min GLP1 vs. placebo, *P* = 0.14). The plasma C-peptide level was suppressed with no difference between sessions, *P* = 0.95. As expected there were no significant differences in circulating hormones and metabolites between sessions. Counter-regulatory hormones rose as expected when PG declined. Insulin levels were similar between sessions, *P* = 0.52 for the treatment effect, as were GH levels, *P* = 0.12. Ghrelin (data not shown), norepinephrine (data not shown), and epinephrine levels were also similar during the sessions, *P* > 0.57. Serum cortisol concentrations were significantly higher with GLP-1 at PG steps of 4.5 and 4.0 mM, *P* = 0.0001 but were similar at PG steps of 3.5 and 3.0 mM, *P* = 0.16 GLP-1 vs. placebo. The glucagon levels increased with placebo infusion at PG step 4 mM and the glucagon levels were increased at PG step 3 mM (significantly so at 270 and 330 min, *P*=0.001 and 0.03, but not 300 and 360 min, *P*=0.14 and 0.05) with GLP-1 infusion. Plasma GLP-1 (total and intact) levels differed between PET sessions, *P* < 0.001.

**Figure 2:**
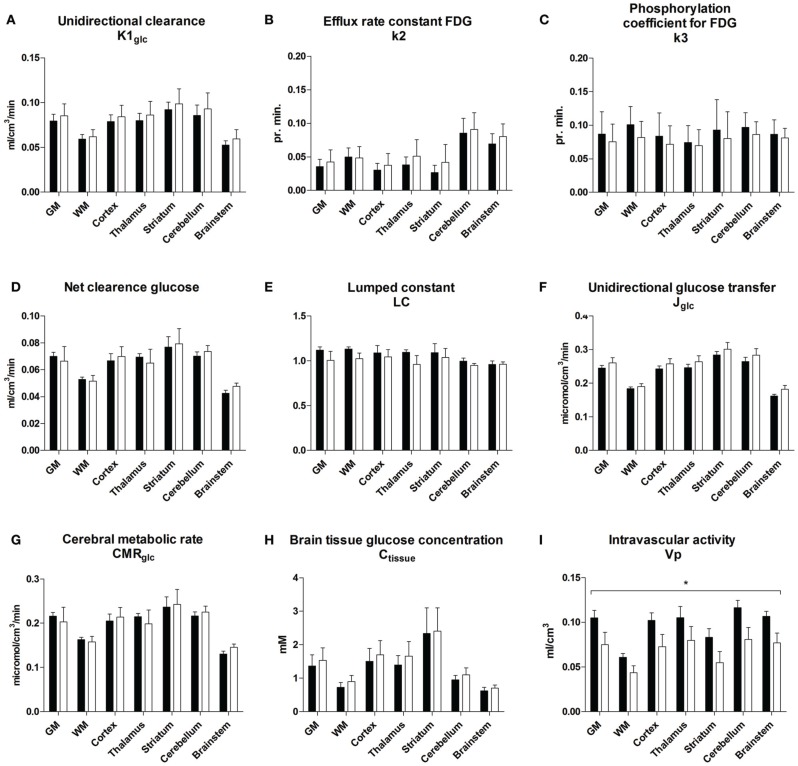
**Unidirectional clearance (A), efflux rate constant for FDG (B), phosphorylation coefficient for FDG (C), net clearance of glucose (D), lumped constant (E), unidirectional glucose transfer (F), cerebral metabolic rate for glucose (G), brain tissue glucose concentration (H), and vascular volume (I) during placebo and GLP-1 infusion.** The regions are total cerebral gray matter (GM), cerebral cortex (Cortex), thalamus (TH), striatum (ST), cerebellar cortex (cerebellum), brain stem (BS) and white matter (WM). Black bars and white bars represent placebo and GLP-1 respectively. Data are mean ± SEM. ^*^P < 0.05. *P*-value for overall GLP-1 effect on *K*_1glc_ = 0.17; *P*-value for overall GLP-1 effect on k^*^_2_ = 0.57; *P*-value for overall GLP-1 effect on k^*^_3_ = 0.48; *P*-value for overall GLP-1 effect on *K*_glc_ = 0.88; *P*-value for overall GLP-1 effect on *LC* = 0.05; *P*-value for overall GLP-1 effect on *J*_glc_ = 0.23; *P*-value for overall GLP-1 effect on *CMR*_glc_ = 0.96; *P*-value for overall GLP-1 effect on *C*_tissue_ = 0.58; *P*-value for overall GLP-1 effect on *V*_*p*_ = 0.003.

The magnitudes of the unidirectional clearance of glucose (*K*_1glc)_ were similar for GLP-1 vs. placebo in all regions (*P* = 0.17, Figure 2A). The efflux rate constant for FDG (k^*^_2_, Figure 2B) did not change significantly (*P* = 0.57) with GLP-1 infusion, compared to placebo. No significant change occurred for the phosphorylation rate constant for FDG (k^*^_3_), *P* = 0.48 (Figure 2C), the net clearances of FDG (K^*^, data not shown), *P* = 0.62 or the net clearances of glucose (*K*), *P* = 0.88 with GLP-1 infusion (Figure 2D). We found a tendency toward decreased lumped constant with GLP-1 infusion in all regions, *P* = 0.05 (*P* = 0.75 for interaction between region and treatment) (Figure 2E). The volume of exchange (*K*_1_/*k*_2_ = K^*^_1_/k^*^_2_) did not change significantly with GLP-1 (*P* = 0.80, data not shown). Unidirectional glucose flux (*J*_glc_) was not significantly affected by GLP-1 infusion in any brain region (*P* = 0.87 for the interaction between region and treatment and, subsequently, *P* = 0.23 for the treatment effect) as shown in Figure 2F. Cerebral metabolic rate for glucose (*CMR*_glc_) remained virtually unchanged in all regions with GLP-1 infusion, *P* = 0.96, as did the cerebral tissue glucose concentration (*C*_tissue_) *P* = 0.58, (Figures 2G,H). The vascular volume in the brain (*V*_*p*_) decreased by 0.03 ml/cm^3^ (95% CI 0.01–0.05) with GLP-1 infusion, *P* = 0.003 with the same effect in all regions (*P* = 0.63) (Figure 2I).

**Figure 3:**
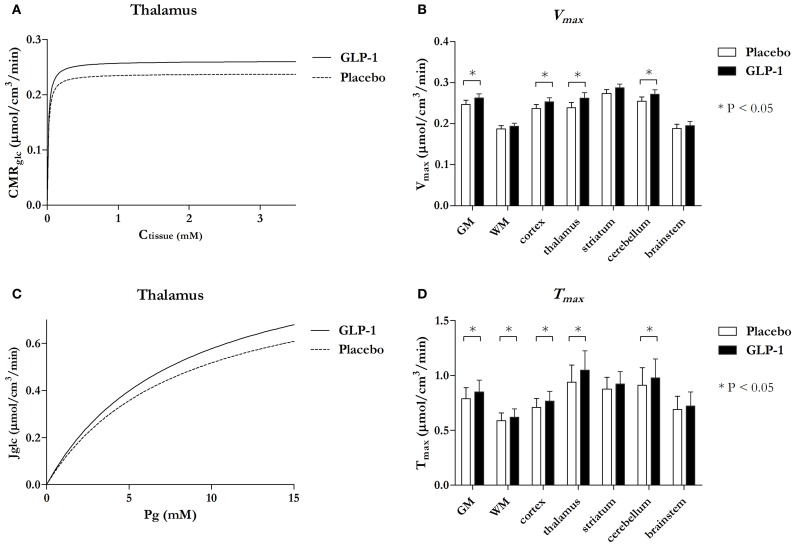
**Michaelis-Menten curves reflecting phosphorylation velocity responding Ctissue in thalamus with and without GLP-1 (A) and Michaelis-Menten curves reflecting unidirectional glucose transport responding PG in thalamus with and without GLP-1 (C) and bar charts comparing maximum phosphorylation velocity *V*_max_ (B) and maximum transport capacity *T*_max_ (D) in the seven regions.** Data in **(A)** and **(C)** are estimates and the bar chart **(B)** and **(D)** are estimates ± SE. ^*^P ≤ 0.05. *P*-value for *V*_max_ in GM = 0.03, cortex = 0.02, thalamus = 0.005, and cerebellum = 0.02. *P*-value for *T*_max_ in GM = 0.025, WM = 0.049, cortex = 0.020, thalamus = 0.004, and cerebellum = 0.040.

When we combined the data with our findings in hyperglycemia (Gejl et al., 2012a), we found that the maximum phosphorylation velocity *V*_max_ increased significantly in several regions with GLP-1 (Figures 3A,B), including GM as a whole (*P* = 0.03), cortex (*P* = 0.02), thalamus (*P* = 0.005), and cerebellum (*P* = 0.02). The difference between the Michaelis-Menten curves of hexokinase activity was not explained by significantly different affinity constants (*K*_*M*_) for the placebo and GLP-1 treatments in any region (*P*-values above 0.22; data not shown). The analysis of the maximum blood-brain glucose transport capacity *T*_max_ with GLP-1 (Figures 3C,D) revealed that *T*_max_ increased significantly in GM as a whole (*P* = 0.025), cortex (*P* = 0.020), thalamus (*P* = 0.004), cerebellum (*P* = 0.040) and white matter regions (*P* = 0.049), and the difference between the Michaelis-Menten curves was not consistent with significantly different affinity constants of the glucose transporter (*K*_*t*_) in the two sessions (*P* values above 0.17; data not shown).

## Discussion

In the present study, we found that effects of native GLP-1 in the brain are dependent on PG concentration as in other tissues, notably pancreatic beta cells (Degn et al., 2004). GLP-1 did not alter brain glucose metabolism or transport during hypoglycemia, contrary to the effect in hyperglycemia (Gejl et al., 2012a). GLP-1 lowered the vascular volume *V*_*p*_ in brain, and, when pooled with data from the hyperglycemia study, we demonstrated increased maximum phosphorylation velocity and increased transport capacity with GLP-1 in specific gray and white matter regions.

Normal brain function depends on a continuous supply of glucose by the cerebral circulation, and blood-brain glucose transfer normally is affected chiefly by changes of the PG level (Gjedde, 1983). Thus, hypoglycemia lowers unidirectional blood-brain glucose transfer as described by the Michaelis-Menten equation. In healthy humans, blood glucose below 3 mM acutely induces brain dysfunction and hypoglycemic symptoms (Maran et al., 1995). Hence the main defense of patients with insulin-treated diabetes is subjective recognition of symptoms such as sweating, hunger, and tremor. An estimated 2–4% of deaths of people with type 1 diabetes have been attributed to hypoglycemia (Cryer, 2004), and hypoglycemia also occurs in type 2 diabetes with prevalence rates of 70–80% in clinical trials of insulin (UKPDS, 1998; Jensen et al., 2010). However, a significant number of these patients are unaware of the hypoglycemia with no warning prior to cognitive dysfunction with severe symptoms such as confusion and coma (Bingham et al., 2005). One report indicates that a relative rise of *CMR*_glc_ occurs in aware subjects during hypoglycemia in contrast to a relative fall in unaware subjects (Bingham et al., 2005). Changes in cerebral glucose uptake have been associated with recurrent hypoglycemic events and are believed to be a risk factor for hypoglycemic unawareness (Boyle et al., 1994; Bingham et al., 2005).

It is uncertain how GLP-1 lowers the *C*_tissue_ during hyperglycemia, but we reason that the most likely effect would be on the *CMR*_glc_ by change of the maximum phosphorylation velocity, e.g., by influencing the activity of hexokinase to a greater extent than the transporter density in the endothelial membranes. If so, GLP-1 could further lower tissue glucose in hypoglycemia.

The current study tested the expectation that the effects of GLP-1 on brain glucose content, blood-brain glucose metabolism, and blood-brain glucose transfer, are additive in hypoglycemia because of the possibly enhanced *CMR*_glc_ and *J*_glc_ noted in hyperglycemia, but the results were not consistent with this prediction. In hypoglycemia, we noted a numerical but statistically insignificant increase of the blood-brain glucose transfer in the presence of GLP-1. The cerebral metabolic rate and the cerebral tissue glucose concentration also remained constant. The results are consistent with the interpretation that the effects of GLP-1 are glucose-dependent, as they are in pancreas. The GLP-1 secretion is preserved during hypoglycemia (Poulsen et al., 2000) and GLP-1 enhances glucose-induced insulin secretion from the pancreatic β-cells via the G-protein coupled GLP-1R leading to an intracellular cAMP increase. Furthermore, GLP-1 inhibits glucagon secretion from the pancreatic α-cells at fasting and elevated glucose concentrations, whereas hypoglycemia induced glucagon secretion is not inhibited—the mechanisms involved are not fully understood (Nauck et al., 2002). Slightly higher glucagon levels during hypoglycemia in the GLP-1 group compared to the control experiments was previously found in studies with the GLP-1 analog exenatide (Degn et al., 2004) and the DPP-IV inhibitor Vildagliptin (Ahren et al., 2009). Glucagon levels do not seem to affect BBB glucose transport or cerebral glucose metabolism (Segel et al., 2001). Judging from the results of both studies, the effect of GLP-1 on blood-brain glucose metabolism and transfer is the result of a combination of increased maximum phosphorylation velocity and unchanged hexokinase affinity, coupled with a significant increase of the transport capacity in most regions and unaltered glucose transporter affinity.

Kinetically, the rates of unidirectional glucose transfer are not rate-limiting for glucose metabolism under normal conditions. However, when PG declines below a certain threshold, the delivery and transport of glucose from blood to brain ultimately fail to support the rate of glucose utilization, with the consequence of impaired brain energy turnover (Pardridge, 1983). The transport capacity is not closely coupled to the metabolic rate but rather influences the balance between glucose delivery and metabolism as reflected in the *C*_tissue_. Changes of *V*_max_ or *T*_max_ with unchanged *K*_*M*_ or *K*_*t*_ or both are therefore important determinants of *C*_tissue_ when the metabolic rate and plasma and tissue glucose increase. Because of the very high affinity of hexokinase to glucose, the increase of *V*_max_ is consistent with an increased metabolism of glucose in response to GLP-1 infusion, depending on the prevailing plasma levels of glucose. The mechanism is speculative; but as the maximum activity of hexokinase appears to be increased, by a GLP-1 mediated activation of the AMP-activated protein kinase (AMPK) is suggested by evidence from an animal stroke model (Hou et al., 2012), with possible increase of metabolism and GLUT1 activity (Ronnett et al., 2009; Cura and Carruthers, 2012). *T*_max_ increased in various regions. Another possible link, published by Mathiisen et al. has shown that astrocytes participate in some functions of the BBB by covering the capillaries (Mathiisen et al., 2010), and therefore astrocytic activity may contribute to the changes observed in response to GLP-1.

Several studies suggest that blood–brain transfer of glucose may be down-regulated in poorly controlled diabetes (Gjedde and Crone, 1981; McCall, 2004). There is some evidence that patients with diabetes with increased PG concentrations may develop cerebral symptoms of hypoglycemia when PG rapidly is lowered to normal concentrations, possibly due to repressive changes of the glucose transport mechanism occurring in the endothelial cells in response to hyperglycemia (Gjedde and Crone, 1981), although the findings are equivocal (Wang et al., 2012). Here, we report opposite results of GLP-1, consistent with increased transport capacity. The elevated transport is of considerable potential interest to the treatment of patients with insulin in whom the co-treatment with GLP-1 might prevent adverse effects from glucopenia.

At the glucose concentrations in blood and brain that prevail in the present study and the previous study, transport is never saturated, whereas phosphorylation by hexokinase is moderately unsaturated in hypoglycemia. In hyperglycemia, phosphorylation is at maximum, although net transport may still change and transfer more glucose across the BBB, raising intracellular glucose without affecting the flux through hexokinase and hence the glycolytic rate.

Blood-brain glucose transfer rarely limits glucose metabolism, but glucose transport across the BBB in principle can be stimulated by (1) increasing the glucose gradient and thereby raising the difference between the glucose fluxes in the two directions across the BBB, (2) increasing intrinsic activity of GLUT-1, or its affinity, or (3) increasing the functionally available GLUT-1 carriers in the BBB by inserting new GLUT proteins with or without increasing the capillary surface area available for facilitated glucose entry (Leybaert, 2005). Together, the latter two mechanisms define the maximum transport capacity (*T*_max_). The results suggest intrinsic changes of the GLUT-1 carrier, or increasing density of GLUT-1 in the BBB, based on similar affinity constants (*K*_*t*_) and decreased plasma volumes in brain (*V*_*p*_) in hypoglycemia and unaltered *V*_*p*_ in the hyperglycemia. Thus we postulate a direct effect of GLP-1 on the GLUT1 properties in the membranes of the BBB, with a possible link by means of AMPK activation.

Cerebral blood flow was not measured in the current study, but the net and unidirectional blood-brain clearances (*K* and *K*_1_) in principle are measures that incorporate blood flow effects, being equal to the product of the extraction fraction (net or unidirectional) and the plasma flow. The clearance in turn yields the substrate flux when multiplied with the substrate concentration.

The trend toward decline of the lumped constant with GLP-1 in hypoglycemia is a further indication of the potentially protective effect of the peptide, because the rise of the lumped constant in hypoglycemia is the obligatory result of the effect of low glucose on the competition between the tracer FDG and native glucose (Gjedde et al., 1985). Hence, a lower lumped constant is an indicator of possibly less perturbed glucose concentration.

We observed a decrease of *V*_*p*_ with GLP-1 infusion. The vascular volume is the part of tissue volume occupied by intravascular blood. Although the degree of hypoglycemia was low in the present study, *V*_*p*_ was still higher than in hyperglycemia, particularly in the placebo group. A similar increase was reported earlier (Kennan et al., 2005). GLP-1 is a peripheral vasodilator in several parts of the body (Grieve et al., 2009; Gejl et al., 2012b), suggesting that the *V*_*p*_ decline could be a sympathetic autoregulatory reaction of the cerebral vasculature to lowered arterial blood pressure. In this context, it is worthwhile to consider the physiology of glucose transport: At a PG of 3.0 mM, the extraction fraction of glucose is not threateningly high (Crone, 1965), but it is close to the threshold where perfusion changes may become important, particularly when PG decreases further. The *V*_*p*_ decrease has no impact on other observations, as *V*_*p*_ refers to the vascular volume and not to the cerebral blood flow. Because of the autoregulatory nature of cerebral blood flow, glucose kinetics remain unaffected.

It is highly debated whether GLP-1 crosses the BBB, or the central effects of GLP-1 and analogues are mediated by leaks through the BBB of the circumventricular organs, and the question remains an area of investigation (Holst et al., 2011), although our results indicate an impact in the brain due to increased metabolism of glucose when glucose is present at normal or higher than normal concentrations.

The dependence of the present analysis on data reported previously raises the issue of comparability of the two experiments, but the analysis of Michaelis-Menten constants requires a wide spectrum of transport and metabolism fluxes vs. glucose concentrations, as obtained in the two studies with the same designs designs in terms of clamp and PET procedure. The participants could not participate in both studies because of the exposure to ionizing radiation, but the demographics were the same. The Michaelis-Menten estimates are based on region-of-interest analysis within the group (hypo- or hyperglycemic) and were not individually calculated.

We conclude that the GLP-1-induced effect on BBB glucose transfer and metabolism in brain, and hence brain glucose content, are glucose-concentration dependent, rendering changes insignificant in hypoglycemia. GLP-1 increases the maximum phosphorylation velocity of glucose without a significant change in affinity of glucose to hexokinase. We found that GLP-1 did affect *T*_max_ in gray matter regions of cortex, thalamus, cerebellum, and white matter regions with unaltered affinity of GLUT1 to glucose. The observation that the effects of GLP-1 on glucose transport and metabolism vanish at low glucose concentrations contributes to a more complete understanding of GLP-1's generally beneficial actions in brain. Thus, under conditions of pharmacologically elevated levels of GLP-1, we note that GLP-1 has no deleterious effect on brain glucose transport in hypoglycemia where glucose availability is low, and thus does not exacerbate the effects of hypoglycemia *per se*.

### Conflict of interest statement

The authors declare that the research was conducted in the absence of any commercial or financial relationships that could be construed as a potential conflict of interest.
